# An Adaptive Classification Strategy for Reliable Locomotion Mode Recognition

**DOI:** 10.3390/s17092020

**Published:** 2017-09-04

**Authors:** Ming Liu, Fan Zhang, He (Helen) Huang

**Affiliations:** Neuromuscular Rehabilitation Engineering Laboratory, UNC/NCSU Joint Department of Biomedical Engineering, North Carolina State University, Raleigh, NC, 27606, USA; mliu10@ncsu.edu (M.L.); fzhang9@ncsu.edu (F.Z.)

**Keywords:** locomotion mode recognition, powered prosthesis leg, adaptive pattern classifier, surface electromyography, and human-in-the-loop

## Abstract

Algorithms for locomotion mode recognition (LMR) based on surface electromyography and mechanical sensors have recently been developed and could be used for the neural control of powered prosthetic legs. However, the variations in input signals, caused by physical changes at the sensor interface and human physiological changes, may threaten the reliability of these algorithms. This study aimed to investigate the effectiveness of applying adaptive pattern classifiers for LMR. Three adaptive classifiers, i.e., entropy-based adaptation (EBA), LearnIng From Testing data (LIFT), and Transductive Support Vector Machine (TSVM), were compared and offline evaluated using data collected from two able-bodied subjects and one transfemoral amputee. The offline analysis indicated that the adaptive classifier could effectively maintain or restore the performance of the LMR algorithm when gradual signal variations occurred. EBA and LIFT were recommended because of their better performance and higher computational efficiency. Finally, the EBA was implemented for real-time human-in-the-loop prosthesis control. The online evaluation showed that the applied EBA effectively adapted to changes in input signals across sessions and yielded more reliable prosthesis control over time, compared with the LMR without adaptation. The developed novel adaptive strategy may further enhance the reliability of neurally-controlled prosthetic legs.

## 1. Introduction

Surface electromyography (EMG) is a commonly used neural control source for many mechanical devices and computers [1]. EMG pattern recognition (PR) is one of the advanced algorithms that can recognize a user’s intention, and has enabled upper limb amputees to control prosthetic joints intuitively and efficiently [2,3,4,5,6]. However, the number of studies that use EMG signals for the neural control of prosthetic legs is relatively limited. EMG PR has recently been designed to interpret either the user's intended motion at lower limb joints [7,8] or the user’s locomotion mode (e.g., level-ground walking and stair ascent) [9,10,11]. For the latter case, a previous study [9] used EMG signals recorded from the residual shank muscles of one transtibial amputee to accurately identify two locomotion modes. Our group developed a phase-dependent EMG PR strategy [10], which can accurately decipher non-stationary EMG signals recorded from two transfemoral amputees during ambulation. The designed classifier based on a linear discriminant analysis (LDA) algorithm exhibited around 90% accuracy in recognizing seven locomotion modes. Furthermore, the performance of the phase-dependent EMG PR algorithm was further improved by introducing a novel neuromuscular-mechanical fusion scheme [8,12]. EMG signals measured from the residual muscles of leg amputees and mechanical signals measured from sensors embedded in prosthetic legs were fused at the feature level. This fusion-based algorithm for locomotion mode recognition (LMR) outperformed the algorithm based on only EMG signals or mechanical signals [8]. It was also observed that the support vector machine (SVM)-based classifier demonstrated an improved performance, compared to the LDA-based classifier. The concept of a fusion-based algorithm has been preliminarily evaluated for the real-time neural control of powered prosthetic legs in recent studies [13,14,15]. With this algorithm, the tested transfemoral amputees were reported to be capable of conducting a seamless transition between different locomotion tasks, such as level-ground walking, slope walking, and stair climbing, in a laboratory environment for a short time evaluation session.

One of the challenges in applying surface EMG signals for the neural control of prosthetics is the degradation of intent recognition performance due to variations of EMG signals over time. Most of the existing intent recognition schemes are static, which means that the system performance totally depends on the muscle activity patterns of each movement during the initial training data collection sessions. Since this non-adaptive scheme cannot accommodate the changes in EMG signals over time, any variations in input signals could degrade the intent recognition performance and influence the system robustness. These signal variations may be caused by physical changes (e.g., electrode shift, baseline noise, or change of impedance) or physiological changes (e.g., human adaptation and muscle fatigue). Here, the human adaptation referred to the procedure of human subjects to gradual change of their motor control strategy for effective interactions with the new prostheses [16]. For example, the location shifts of electrodes may result in magnitude changes of EMG signals [17,18,19]; muscle fatigue causes drift in EMG medium frequency and magnitude [20,21]. One straightforward solution is system recalibration; however, a frequent recalibration procedure is time-consuming which makes it impractical for the clinical use of prosthetics. Several other advanced solutions have also been explored, such as the selection of robust EMG features [20,22], special training strategies [23], sensor fault detection [24], and adaptive pattern recognition strategies [25,26,27,28,29,30].

Several adaptive algorithms [25,26,27,28,29,30] have been reported to improve the robustness of recognizing the user’s intended upper limb joint motions. Generally, adaptive algorithms include supervised approaches [25,27,30], in which the user’s intended locomotion mode (correct class) is known; and unsupervised approaches [26,28,29], in which the user’s intended locomotion mode (correct class) is unknown.

Most of these previous studies focused on upper limb cases; however, the design of an adaptive LMR algorithm towards the reliable neural control of powered prosthetic leg is still limited and needed. This is because the architectures of LMR algorithms are different from those used for recognizing intended joint motions for upper-limb prostheses. In addition, the demand of reliability is higher for the operation of lower limb prostheses than upper limb devices, because the unreliable performance may cause the use to fall and threaten the safe and confident use of lower limb prostheses. To the best of the authors’ knowledge, only one other group has demonstrates an initial effort to generate an adaptive LMR [30], which has not yet been adopted for any amputee patient.

Motivated by the need for a reliable PR algorithm for locomotion mode recognition, this study investigated and evaluated the effectiveness of adaptive PR algorithms in improving the robustness of locomotion mode recognition over time. In Part 1, three adaptive algorithms were applied and offline evaluated based on the experimental data collected from able-bodied subjects and unilateral transfemoral amputees. In Part 2, the most effective adaptive algorithm was initially implemented for real-time human-in-the-loop prosthesis control and online evaluated for one transfemoral amputee. To the best of our knowledge, this is the first attempt where the adaptive LMR algorithm was implemented and evaluated on powered prosthetic leg for online control for amputees. The outcomes of this study are expected to provide a potential solution for the future design of a reliable neural-machine interface, which makes the neural control of powered prosthetic leg practical and reliable.

## 2. Part 1: Investigation of Effectiveness of Different Adaptive Pattern Recognition Algorithms

### 2.1. Method and Materials

#### 2.1.1. Experiment Participants and Data Collection

This study was approved by the Institutional Review Board (IRB) at the University of North Carolina at Chapel Hill and all the subjects gave their consent. Two able-bodied subjects (AB01 and AB02) and two unilateral transfemoral amputees (TF01 and TF02) were recruited in this study. AB01, AB02, and TF01 participated in the experiment of Part 1; and TF02 was tested in the experiment sessions of Part 2. The demographic information of all the recruited subjects is summarized in Table 1. The recruited AB subjects had no history of orthopedic or neurological pathologies. Two amputee subjects were both regular passive prostheses users in daily life. During the experiment, the subjects were instructed to walk with a prototypical powered prosthetic leg developed in our lab [31].

The powered prosthetic leg consisted of a powered knee and a passive ankle. The device was controlled through a hierarchical control structure. The LMR system identified the current locomotion mode (such as W, RA, and RD). A set of predefined control parameters were assigned based on the identified locomotion mode. Based on the assigned control parameters, the power prosthesis leg was controlled by a finite-state impedance controller, which separated each gait cycle into five phases. More details about the mechanical design and control methodology of our powered prosthetic leg can be found in [31].

Each subject went through a tuning procedure to get used to the powered prosthetic leg. During the tuning procedure, subjects were instructed to walk at a fixed speed on a treadmill, to walk on the level ground with a self-selected speed, and to walk on ramps and stairs. During the tuning sessions, the prosthesis control parameters were customized for each subject by a tuning expert and the gait performance during different locomotion tasks was validated by a certified physical therapist. Amputee subjects usually finished this procedure quicker when compared to able-bodied subjects.

**Figure 1:**
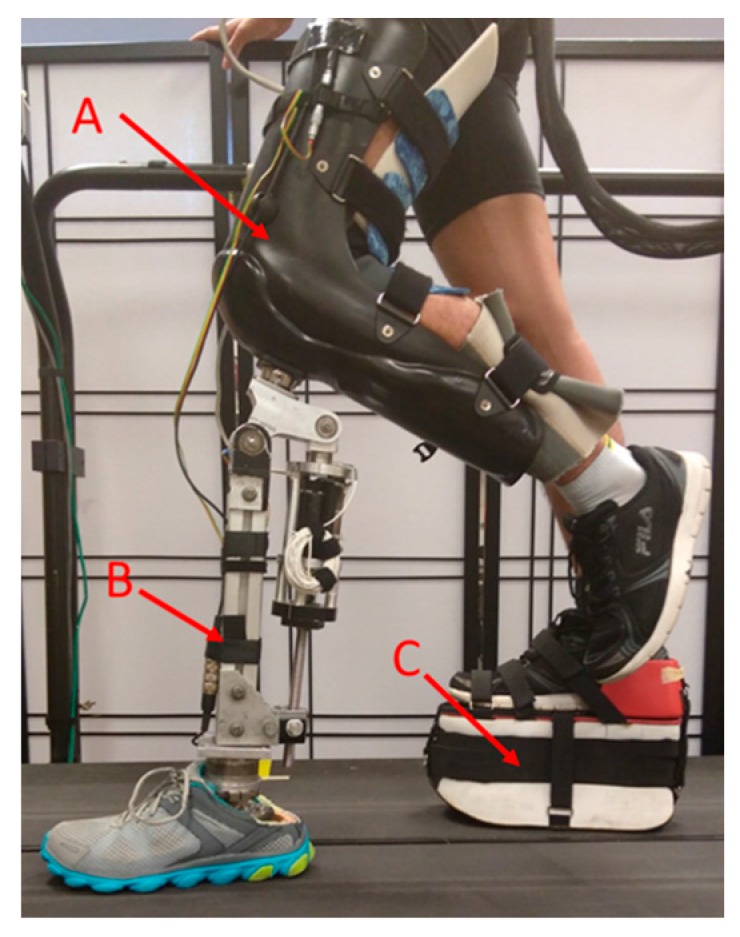
An able-bodied subject wearing the powered prosthetic leg. A was an abled-bodied adaptor, B was the powered prosthetic leg, and C was a foot raiser, which was used to ensure the length of the prosthetic leg and the unaffected leg was the same.

For AB subjects, a special designed L-shaped adaptor was constructed, so that they could walk with the powered prosthetic leg (as shown in Figure 1); for transfemoral subjects, a suction prosthetic socket was customized for each individual.

Seven EMG signals were recorded from targeted thigh muscles, including rectus femoris (RF), vastus lateralis (VL), vastus medialis (VM), biceps femoris long head (BFL), semitendinosus (SEM), tensor fasciae latae (TFL), and biceps femoris short head (BFS). For able-bodied subjects, electrodes were placed based on anatomical locations [32] and the muscles were palpated. For transfemoral subjects, due to the lower limb amputation and scar tissues on the residual limb, the EMG locations were approximated and guided by checking EMG recordings and palpations when the subjects were instructed to perform hip motions and imagine and execute knee flexion/extension. A ground electrode was placed on the anterior iliac spine. A 16-channel EMG system, MA300-XVI (Motion Lab System, Baton Rouge, LA, USA), was used to collect bipolar EMG signals with EMG electrodes at a 22 mm inter-electrode distance. The EMG electrodes contained a preamplifier that band-pass filtered the EMG signals between 10 and 2000 Hz with a pass-band gain of 20. For transfemoral subjects, the electrodes were integrated into the experimental suction sockets to ensure reliable electrode-skin contact and the user’s comfort after donning the sockets. In addition to EMG measurements, mechanical ground reaction forces/moments were recorded using a six-degree-of-freedom (DOF) load cell (Mini58, ATI, Apex, NC, USA) mounted on the prosthetic pylon. Both EMG and mechanical measurements were synchronized and sampled at 1000 Hz. Additionally, the powered prosthesis status (i.e., prosthesis modes and gait phases) was also recorded.

#### 2.1.2. Experimental Protocol

In the experiment, our subjects were instructed to perform multiple locomotion tasks with the powered knee prosthesis in a laboratory environment. Five common locomotion tasks in daily life were considered, including level-ground walking (W), ramp ascent/descent (RA/RD), and stair ascent/descent (SA/SD). In addition, eight task transitions were tested in this study: W→SA, W→SD, W→RA, W→RD, SA→W, SD→W, RA→W, and RD→W. For level-ground walking, the subject was asked to walk on a 4.57 m straight walkway; for ramp ascent/descent, the subject walked on a 3.05 m ramp with a 10-degree inclination angle; for stair ascent/descent, the subjects were required to negotiate an eight-step staircase with a stair height of 13.5 cm. The subjects seamlessly transitioned between different locomotion modes, as an experienced experimenter manually switched the control modes of the prosthesis at the critical timing defined in our previous study [8].

The experiment included two sessions: an initial training session and testing session. In the initial training session, a training data set used to build the pattern classifier was collected. The subjects were instructed to perform all the studied locomotion modes continuously in a sequential order (i.e., W→SA→W→SD→W→RA→W→RD→W). The performance of these sequential tasks was repeated three times. Then, in the testing session, the subjects completed 10 trials, in which the same sequence of locomotion tasks was included. A fall-arrest harness system and hand rails were adopted to ensure the safety of the subjects. To avoid fatigue, subjects were allowed to sit for three minutes between trials.

Prior to the experiment, each subject only received a limited amount of gait training on how to walk with the experimental powered prosthetic leg. The purpose of this experimental design was to introduce realistic changes in input signals caused by the gait variations in the initial human-prosthesis adaptation process. Based on our previous observation [31], when lower limb amputees or able-bodied subjects walked on the powered knee prostheses for the first time, their gait pattern (including kinematics, kinetics, and EMG signals) gradually varied over time. This was because the prosthesis user had to re-learn how to walk with the powered prosthetic leg, which redefined the dynamics of the human-prosthesis integrated system. Usually, gait training was necessary so that lower limb amputees could adapt to the powered prosthetic leg and generate consistent walking patterns, which were important to ensure reliable LMR. In this study, however, we captured the greatest extent of input signal variations on the part of the subjects during the initial adaptation and evaluated the effectiveness of adaptive algorithms against these realistic signal changes.

#### 2.1.3. Locomotion Mode Recognition Algorithm

**Figure 2:**
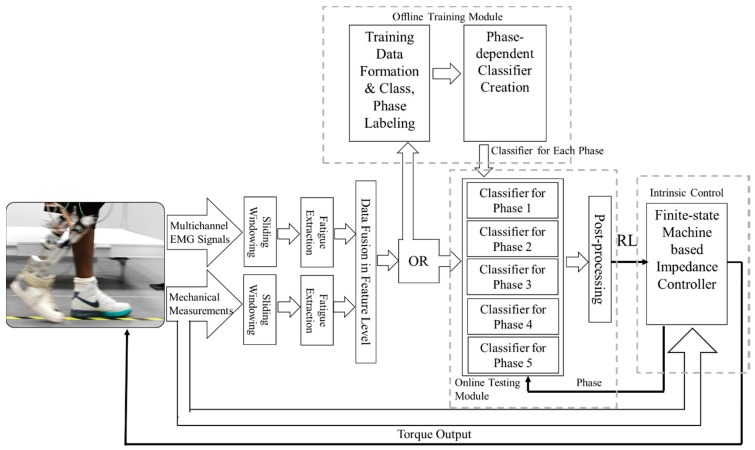
The locomotion recognition system used for lower limb prosthesis control. RL: recognized locomotion model.

**Figure 3:**
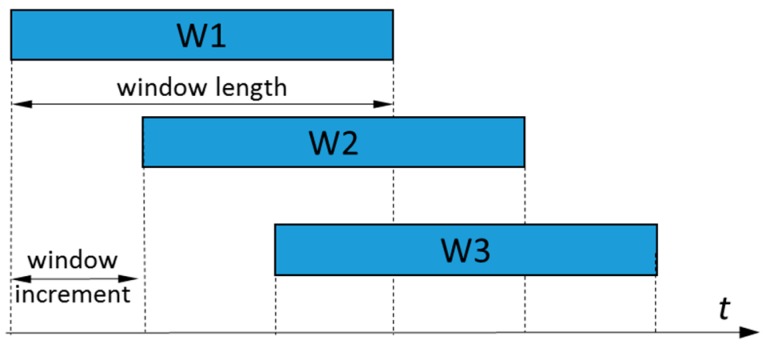
The moving windows used to calculate the features.

Phase-dependent LMR based on neuromuscular-mechanical fusion has been developed and tested in our lab [8] (as shown in Figure 2). In this study, all the investigated adaptive schemes and non-adaptive algorithms were implemented upon this algorithm. EMG and mechanical measurements were first segmented into a sliding analysis window with a 160 ms window length and a 20 ms window increment (see Figure 3 for more information about the definition).

Within each window, we band-passed the EMG data using a 20–420 Hz eighth-order Butterworth digital band-pass filter. Then, four time domain features were extracted from each window: the average absolute value, signal length, and number of slope sign changes and zero crossings. Among them, the average absolute value was the average of the absolute value of the EMG signal in the window; the signal length was the cumulative length of the EMG signal in the window; the number of slope sign changes represented how often the slope of the EMG signal changed; and zero crossing was the number of times that the EMG signal crossed the zero amplitude level. For the mechanical signals, the maximum, minimum, mean values, and standard deviation were extracted as the features. More information on the feature calculation can be found in [19,20].

Then, the EMG and mechanical features were fused into one vector and sent into one of the five phase-dependent classifiers, which were each associated with one gait phase, respectively. To identify the locomotion modes, a multiclass SVM [33] with a “one-against-all” (OAA) structure and radial basis kernel function (RBF) was used as the pattern classifier. Detailed parameters of each classifier were decided using data collected in the training sessions. A majority vote was used as the post-processing to generate more reliable classification results. The labeling of locomotion modes (classes) and gait phases was completed by examining the prosthesis status (i.e., control modes and gait phases) recorded during the experiment. Definitions and transition rules of the gait phases were described in [31].

#### 2.1.4. Adaptive Classification Strategies

Three commonly used unsupervised adaptive algorithms were evaluated and compared in this study, including an entropy-based algorithm [27], LearnIng From Testing Data (LIFT) [34] algorithm, and Transductive SVM (TSVM) [35]. These three adaptive schemes were applied on the neuromuscular-mechanical fusion-based LMR algorithm described in Section 2.1.3.

A. Entropy-based Adaptation Algorithm

The entropy-based adaptive algorithm has been investigated and found to be effective in several EMG-based neural-machine interfaces for artificial arm control [25,26,27]. Entropy, defined as (1), is a measure of confidence for classification decisions [27].

(1) $$E\left(n\right)=-\sum_{k=1}^{N}p_{k}\left(n\right)\ln\left[p_{k}\left(n\right)\right]$$

wherein, $p_{k}\left(n\right)$ is the conditional probability of the *n*th decision for the class *k* out of *N* classes (*N =* 5 in this study). Here, $p_{k}\left(n\right)$ was the estimated posterior probability from the SVM outputs using the method of J. Platt. More details about estimating the posterior probability can be found in [36]. A low-entropy decision indicates that one is highly confident and mostly leads to a correct classification; while a decision with a high entropy value indicates low confidence. By monitoring the entropy of each classification decision, the entropy-based adaptive algorithm chooses the testing data with low-entropy decisions to augment the retraining data set, and periodically updates the classifier.

To evaluate this adaptive algorithm in our application, EMG and mechanical features collected in the initial training session were first adopted as the training dataset to train the pattern classifiers. Then, the classifiers were applied to the data collected during the first testing trial for locomotion recognition. The features associated with the decisions, which were given low entropy values, were then added to the training dataset to re-train the classifiers. The updated classifiers were then applied to the features collected in the next testing trial to evaluate the recognition performance. This procedure ran repeatedly until all of the testing trials were evaluated. In this study, the applied threshold for the low entropy value was selected at 0.6, which was optimized in our previous study [37]. In this study, the applied threshold for the low entropy value was selected at 0.6, which was optimized [37] by scanning the potential threshold value between 0.01 and 1.6 based on pilot data collected when subjects used their own prosthetic leg. Then, we identified the value, which maximized the classification accuracy of these pilot data.

B. LearnIng From Testing data (LIFT)

The main principle of LIFT is to use multiple binary classifiers to label the testing data and augment the training data adaptively based on their labels. LIFT was selected because it can handle the case in which the number of training data was relatively limited compared to the amount of testing data [34], which was suitable for our application.

**Figure 4:**
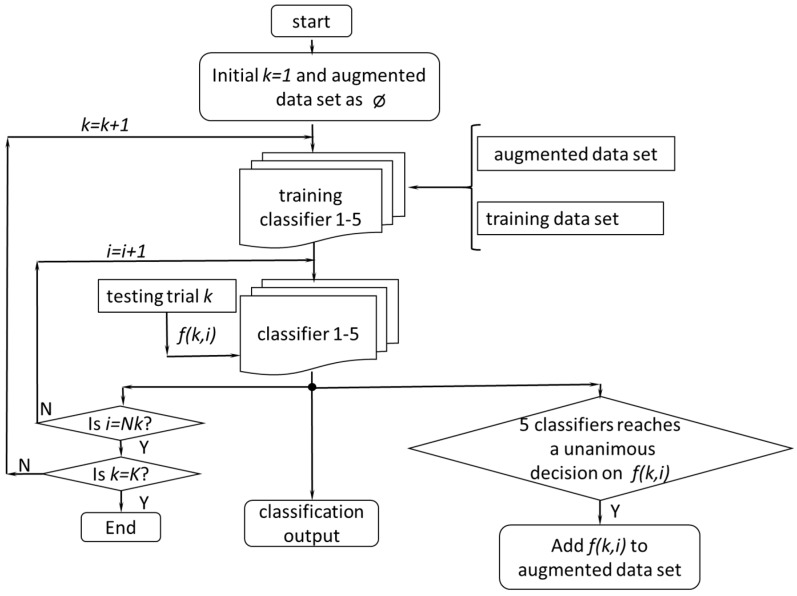
The flow chart for the LIFT. *K* was the total number of testing trials; *Nk* was the number of features collected from the *k*th trial; and *f*(*k*,*i*) represented the *i*th feature in *k*th trial.

Since five locomotion modes were considered in this study, five binary SVM classifiers were formulated based on the initial training dataset. The LIFT procedure is shown in Figure 4. After the initialization, the classifiers, which were of the OOA, were trained by the training data set. In the first iteration, these five classifiers were applied to the data in the first testing trial and outputted five binary decisions for each feature vector *f*(*1*,*i*) (calculated in each analysis window). Because each classifier represented a locomotion mode, the classifiers reached a unanimous binary decision, if only one of the classifiers made a decision that this feature was in the locomotion mode, which was represented by the classifier. If such a unanimous binary decision was made on one feature vector, the feature vector was selected as augmented data and was added to the augmented data set. After all the samples in the first testing trial were tested, the augmented data set and the training data set were used to re-train the classifier. In the second iteration, the newly updated classifier was applied to the next testing trial and the same procedure was repeated. This process was repeated until the last testing trial had been completed. A more detailed description can be found in [37].

C. Transductive Support Vector Machine (TSVM) Adaptation Algorithm

TSVM is specifically designed for SVM classifiers and can be used for both labeled and unlabeled data to build a robust learning model. This algorithm has been successfully applied in many applications, including brain-machine interfaces [38].

**Figure 5:**
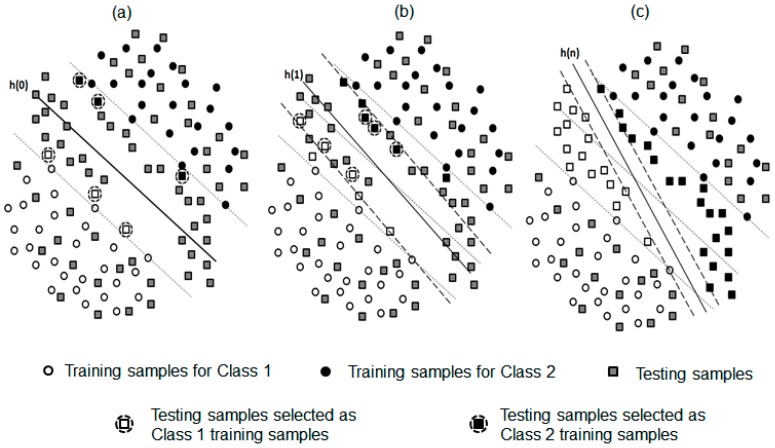
Concept of the TSVM adaptive algorithm for a binary classification problem. Classification hyperplane h (solid line) and margin bounds (dashed lines) in a two-dimentional feature space were shown at the (**a**) first iteration, (**b**) second iteration, and (**c**) last iteration. The dashed circles highlighted the testing samples selected as training data for Class 1 and Class 2, which were called transductive data.

For a better demonstration, the procedure of the TSVM adaptive algorithm with respect to a binary problem is demonstrated in Figure 5. The initial decision hyper-plane (*h*^(0)^) was built based on the features collected in the initial training session (Figure 5a). The margin boundaries for Class 1 and Class 2 (indicated by the black dash line) determined the margin width. The testing data (depicted as a square marker) were projected into the feature space. To select the transductive testing samples used for updating the decision hyper-plane, two criteria were applied: (1) the selected samples must affect the position of the decision hyper-plane *h* to update the training dataset (i.e., the distance to $h^{\left(0\right)}$ is between 0 and 1), and (2) the distance from the selected samples to the hyper-plane is close to 1. Examples of such selected transductive samples (i.e., the testing samples with a dashed circle) were shown in Figure 1. In each iteration, the number of selected transductive samples for each of the two classes was a constant integer (i.e., 5 in this study). Then, the selected transductive feature samples were removed from the testing dataset and used as new training samples to build the hyperplane $h^{\left(1\right)}$, as shown in Figure 5b. This procedure was repeated until all the unlabeled testing samples were assigned to a class (demonstrated in Figure 5c). When applying this procedure to a multi-class problem, we used an OAA classification strategy [35]. The decision hyper-planes in five binary classifiers were iteratively updated after each testing trial.

#### 2.1.5. Evaluation of Effectiveness of Adaptive Strategies

We evaluated the performance of the adaptive LMR systems in static states and transition periods, respectively [8]. Here, static states indicated that subjects continuously performed a single locomotion mode. In the static states, the classification accuracy was adopted as an evaluation metric. The transitional period indicated that subjects changed the locomotion mode, which included a full gait cycle and two stance phases of the powered prosthetic leg. The transitional period started at the initial prosthetic foot contact before stepping on a new terrain and terminated after the single stance phase ended in the next gait cycle. For transition periods, the number of missed transitions was calculated [8]. A missed transition was labeled if the task transition was incorrectly recognized at the end of the transitional period.

### 2.2. Results for Part 1

**Figure 6:**
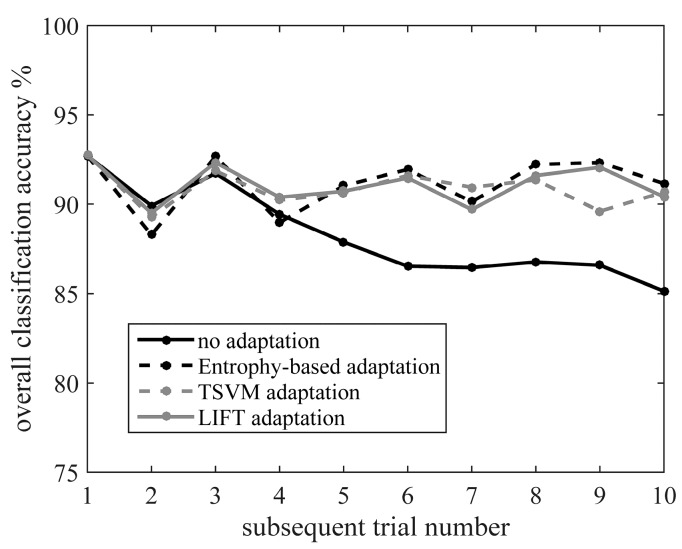
Classification accuracy over time when using three different adaptation strategies and a non-adaptive classification scheme. The accuracy curves were generated from the experimental data collected from TF01.

Figure 6 shows the classification accuracy of static states over time when three different adaptive algorithms were applied to the data collected from TF01. In comparison, the over time performance of the non-adaptive algorithm (i.e., classifier built purely based on the initial training data) was also demonstrated. When the non-adaptive algorithm was used, the classification accuracy (indicated by solid black line in Figure 6) decreased from 92.7% (i.e., in the first testing trial) to 85.1% (i.e., in the last testing trial). In contrast, all the adaptive algorithms were able to effectively maintain the accuracy at around 90% throughout all the testing trials, even though variations in the input signals occurred over time. A similar performance was also observed for AB01 and AB02.

**Figure 7:**
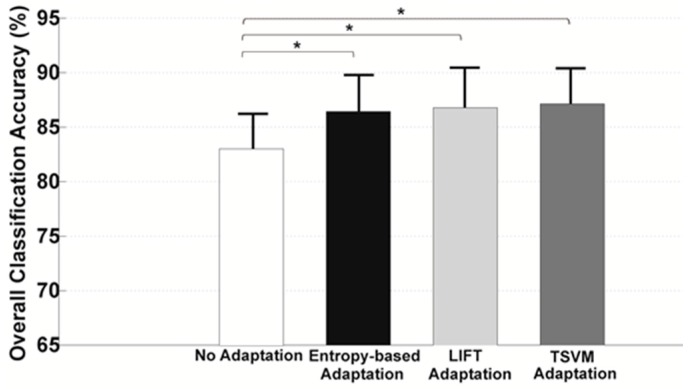
Classification accuracy averaged across the last five testing trials and across all the tested subjects. * indicated a statistically significant difference (one-way ANOVA, *p* < 0.05).

To better demonstrate the improved reliability of the adaptive algorithms over the non-adaptive algorithm, the averaged classification accuracy in static states across the last five testing trials was shown in Figure 7. The accuracy was averaged across AB01, AB02, and TF01. It can be seen that all the adaptive strategies produced a significantly higher accuracy (approximately 3.5–4.3% increase in classification accuracy) than the non-adaptive algorithm (one-way ANOVA, *p* < 0.05). However, no significant difference in classification accuracy was observed among entropy-based, LIFT, and TSVM algorithms (one-way ANOVA, *p* > 0.05).

The locomotion mode recognition performance in the transition period for each tested subject and applied algorithm is summarized in Table 2. Without the adaptive strategy, eight to 12 out of 80 missed task transitions were observed; whereas, only three to six task transitions was missed if adaptive algorithms were applied.

### 2.3. Discussion about Part 1

In this part, we investigated the performance of three different adaptive algorithms in improving the reliability of LMR against variations in input signals. To evaluate the performance of the adaptive algorithms, realistic slow changes in input signals (i.e., EMG signals and mechanical measurements) were experimentally introduced when the subjects were adapting to the powered knee prosthesis for the initial use. It was observed that the locomotion mode recognition performance gradually deteriorated over time if the non-adaptive algorithm was applied. The adaptive pattern classification strategy demonstrated great capability in maintaining the LMR accuracy over time, even when gradual variations of input EMG and mechanical signals occurred. This also implied that the cyber adaptation (i.e., adaptation in pattern classification) was potentially efficient in addressing the unreliability of locomotion mode recognition caused by gradual input signal variations.

Among the three studied adaptive strategies, the entropy-based and LIFT adaptation were suggested for reliable LMR over the TSVM method, due to (1) their satisfactory performance and (2) their relatively low computational complexity. All three adaptive methods presented a comparable classification accuracy in static states when gradual realistic signal variations were introduced. However, during the task transition period, especially for the subject TF01, the entropy-based and LIFT adaptive approaches generated a lower number of missed transitions than TSVM. This might be because TSVM used a fundamentally different adaptation mechanism from the entropy-based algorithm and LIFT algorithm. The latter two methods directly selected the testing samples that produced confident decisions as the new training data to update the classifier, while the TSVM adaptation was expanded from the basic concept of SVM, which shifted the decision hyperplanes by iteratively inserting a pairwise set of positive and negative testing (i.e., transductive) samples into the training data. A previous study [39] also reported that it became difficult for TSVM to complete the adaptation if the pattern distributions of the original training features and new testing features were considerably different. In terms of complexity in designing and implementing these three adaptive PR algorithms, TSVM is the most complicated algorithm, which involves iterative boundary adjustments in each adaptation step for an optimal classification performance. Additionally, since the TSVM algorithm is derived from the SVM classifier, the base classifier is constrained to SVM only, which limited the generalization of this approach. Nevertheless, the entropy-based approach and LIFT algorithm are relatively simple to compute, easy to design, and flexible to use. Therefore, in Part 2 of this study, only the entropy-based adaptive algorithm was selected as a representative method for the implementation and online evaluation of the real-time prosthesis human-in-the-loop control.

Although the adopted testing cases in this study only represented a short procedure compared to the usage life of a prosthesis, the capacity to improve the performance in the testing cases was still a good indicator that adaptive classifiers could improve the performance of the prosthesis control in general. Based on our experimental design, the adopted testing case represented the procedure, in which an amputee was exposed to a new prosthesis. Although this procedure was short compared to the whole usable life of a prosthesis, amputees were expected to experience additional variation beyond the one caused by small shifts of electrodes and changes of temperature and humidity. Additional variation brought a higher level of challenges to prosthetic controllers. Demonstrating the capacity to maintain a high performance in such a challenging scenario enabled us to believe that these adaptive classifiers were capable of handling routine variations, which subjects encountered in everyday life.

Unlike many upper limb studies, able-bodied subjects did not outperform amputees in this research. This performance similarity was not a surprise because of the additional challenges for able-bodied subjects to maintain a prosthetic gait. Our locomotion recognition system relied on signals from both EMG sensors and mechanical sensors embedded in the powered prosthesis. To use this locomotion recognition system, the subjects needed to reproduce a natural muscle firing pattern and maintain a relatively smooth prosthetic gait pattern on each terrain. Although able-bodied subjects controlled their muscles well, they needed additional efforts to walk with a prosthetic gait. On the other hand, amputees had little difficulty generating a prosthetic gait, but they had difficulty in recruiting some of their muscles on the residual limb. As a result, the amputees and able-bodied subjects exhibited relatively similar performances when using the locomotion recognition system.

## 3. Part 2: Online Evaluation of Adaptive Locomotion Mode Recognition for Reliable Prosthesis Control

### 3.1. Methods and Materials

#### 3.1.1. Participant and Experimental Setup

TF02 participated in the experiment of Part 2. The demographic information of TF02 is shown in Table 1. Experimental measurements (i.e., EMG signals and mechanical ground reaction forces/moments from prosthesis) and instrumentations were the same as those described in Part 1. In contrast to Part 1, in which the prosthesis tasks was manually switched by an experimenter, the control tasks (i.e., W, RA, RD, SA, SD) of the powered prosthetic leg was modulated by the output of the LMR algorithm (i.e., recognized prosthesis user’s locomotion intention) in Part 2. Both the entropy-based adaptive algorithm and the non-adaptive algorithm were online implemented and hierarchically integrated with the impedance controller of the experimental powered prosthetic leg for real-time neural control. Throughout the entire experiment, the subject was protected by a fall-arrest harness system and hand rails.

#### 3.1.2. Experimental Protocol

Prior to the experiment, TF02 went through a tuning session to become familiar with the powered prosthetic leg. The tuning purpose was to identify personalized control parameters and to allow the amputee to get used to the powered prosthetic leg, since it was different from his daily-used passive device. TF02 was able to acclimate to the powered prosthetic leg and maintain a stable and consistent gait performance at the end of the tuning sessions.

**Figure 8:**
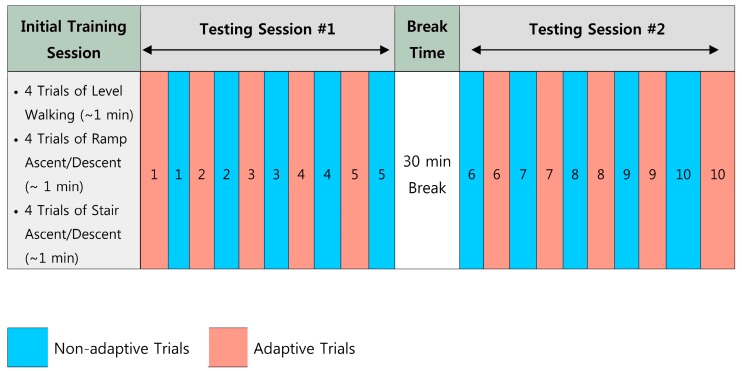
Illustration of experimental protocol design for evaluating adaptive locomotion recognition on online prosthesis control.

The experimental protocol design is illustrated in Figure 8. At the beginning of the experiment, an initial training dataset was collected in a training session for initial classification training. The subject was asked to perform four trials of W, RA, RD, SA, and SD, respectively. Each locomotion task lasted for about 1 min. The online evaluation testing session was divided into two sub-sessions: testing session #1 and testing session #2. In each testing session, the adaptive and non-adaptive trials were alternated in the sequence shown in Figure 4. This sequence of the testing trials was blinded to the subject. In non-adaptive trials, the classifier based purely on the initial training dataset was evaluated and the classifier was never updated. At the end of each adaptive trial, the data associated with low-entropy decisions were added to augment the training dataset. The classifier was re-trained based on this newly updated training dataset and applied to the next adaptive testing trial. In total, we conducted 10 adaptive and 10 non-adaptive testing trials. To ensure the online processing capacity, the window increment was increased to 50 in this online experiment.

There was a 30-min rest period between two sub-sessions. During the rest period, the subject was asked to don and doff the experimental EMG socket. In addition, the room temperature was increased from 19 °C to 23 °C. The purpose of this procedure was to purposely introduce realistic variations in EMG signals by (1) slightly shifting the EMG electrode locations, and (2) changing the temperature and moisture in the skin-socket interface. Although the impact of the do-on and do-off procedure on the location of the electrodes and humidity in the socket was well expected, we did not measure these changes quantitatively due to the challenge of conducting accurate measurements in the socket after the amputees put on their sockets.

#### 3.1.3. Evaluation Approaches

In addition to the evaluation metrics (i.e., classification accuracy in static states and number of missed transitions) described in Part 1, the subject’s prosthesis walking stability during the testing session was also quantified. A handheld manual trigger device was designed for the subject to report his subjective feeling of gait stability. Once he felt unstable, he was asked to push the trigger button. This signal was recorded and synchronized with other experimental measurements. The whole experiment was video-taped.

### 3.2. Results of Part 2

**Figure 9:**
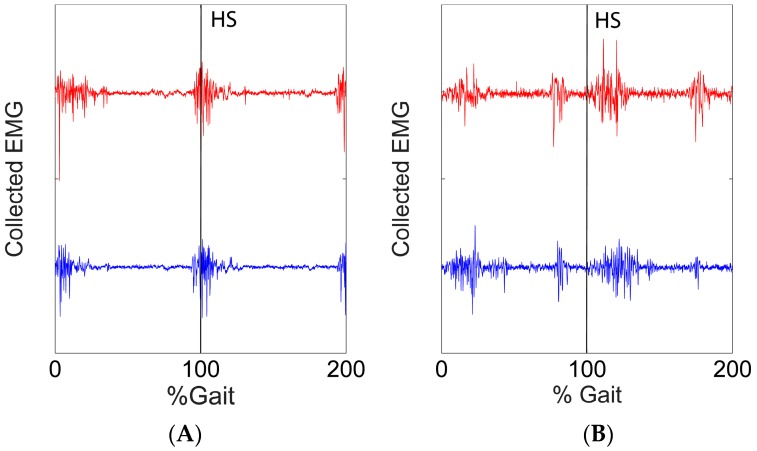
Comparison of raw EMG signals from two channels (Channel (**A**) BFL and Channel (**B**) RF) at the training session and at the final test session (adaptive #10). The EMG signals were collected in two gait cycles at level ground walking. The red line showed the EMG at the training session and the blue line showed the EMG at the final test sessions. The vertical lines indicated heel strike (HS).

Variation of the EMG signals were observed during the experimental procedure. In Figure 9, we show the variation in the EMG signals from two EMG channels (BFL and RF) when the subject was walking on the level ground. The red line and the blue line showed the signals collected in the training session and in the last test sessions (adaptive #10) respectively.

**Figure 10:**
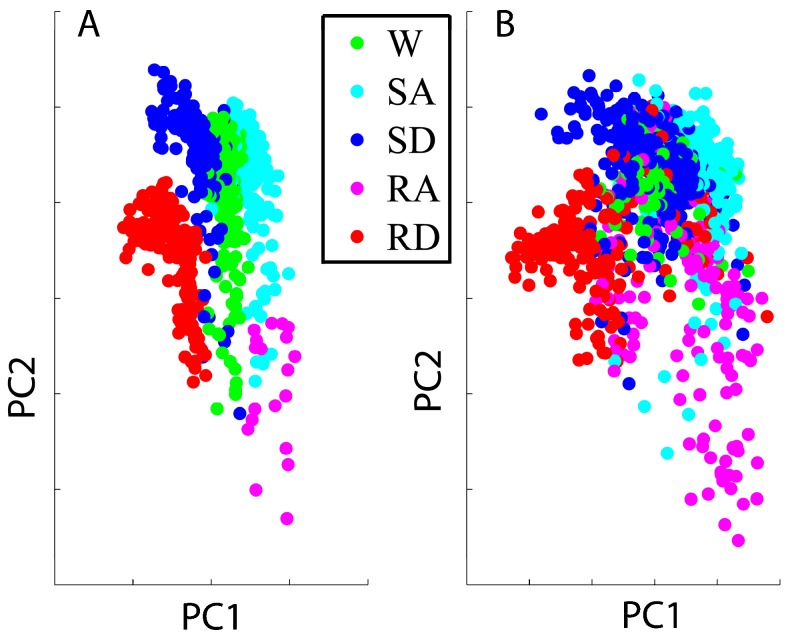
The distribution of features in the feature space, which projected along the first two principal components (PC1 and PC2). The orientations of the principal components were decided by the features from the training session. (**A**) Features are based on the data collected in the initial training session and (**B**) features are based on the data collected in the adaptive trial #10.

Variation of the EMG signals also affected the distribution of features in the feature space. The features based on the initial training session and the adaptive trial #10 were projected into a component space defined by the first two principal components, which were calculated based on the features from the training session. The projections are shown in Figure 10A,B, respectively.

**Figure 11:**
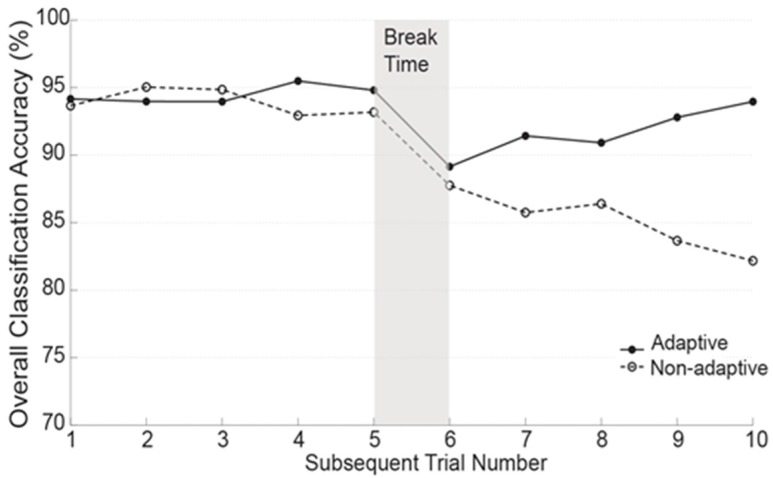
Classification accuracy in static states over time when using the entropy-based adaptive scheme and non-adaptive algorithm for real-time prosthesis control.

Figure 11 demonstrates the locomotion mode recognition accuracy in static states in adaptive and non-adaptive trials over time. In testing session #1 (Trial 1–5), the accuracy in adaptive and non-adaptive trials was very close and stable, ranging from 93.5 to 95.2%. After the break time, the accuracy in both adaptive and non-adaptive trials dramatically dropped below 90% in trial 6. In the following trials, the adaptive algorithm was capable of recovering the LMR performance and restored the accuracy back to 94.3% in the last testing trial; however, the accuracy in the non-adaptive trials kept deteriorating and decreased to 82.4% at the end of the session.

Table 3 lists the number of missed task transitions and the number of unstable disturbances caused by the locomotion recognition errors in adaptive and non-adaptive trials. The number of unstable disturbances was counted as the number of times that the subject pressed the trigger device. In the non-adaptive trials, a total of seven task transitions was missed; whereas in the adaptive trials, only two missed task transitions were observed. These two missed cases happened in trial 6 when the subject transitioned from W to RA and RD. In non-adaptive trials, there was a total of four times in which the subject’s stability was disturbed by the recognition errors, for which three happened on SD and one occurred on RD. All of these gait instability cases were reported in testing session #2. In contrast, the subject was able to safely ambulate without reporting any unstable case during the adaptive trials.

The average processing time for one decision in each trial was 44.2 ± 3.7 ms when executing the algorithm in MATLAB by running it on a desktop computer (Dell Precision T3800 with 2.8 GHz E5-16030 CPU and 4 GB RAM). The same computer also controlled the prosthetic leg.

### 3.3. Discussion for Part 2

In this study we demonstrated the potential of adaptive LMR algorithm for online powered prosthetic leg control. Most of the current adaptive pattern recognition strategies were developed towards the neural control of upper limb prostheses [25,26,27,28,29]; very limited research has been focused on the application of lower limb prosthesis control. In addition, the focus of these upper limb studies has been on either offline error rate analysis or online performance evaluation without an actual prosthesis involved [25,26,27,28,29]. In our study the adaptive LMR was implemented and evaluated on prostheses with human-in-the-loop. Beyond classification accuracy, we also adopted outcome measurements, such as sense of instability, to quantify the effects of LMR errors on human-prosthesis systems. We believe that the outcomes of our study could potentially benefit the future design of reliable neural control for powered prosthetic legs.

Our online testing results also indicated that the entropy-based adaptive algorithm can effectively accommodate the changes in EMG signals and quickly recover the locomotion mode recognition performance across online testing sessions. As shown in Figure 11, the adaptive and non-adaptive algorithms generated a relatively comparable recognition accuracy in testing session #1 (Trial 1–5), possibly because the changes in EMG signals in the first several trials were small. Signal changes were introduced during the break period, potentially causing electrode location shift and inner socket environment changes. The accuracy in both the adaptive and non-adaptive trials suddenly decreased below 90% in trial 6, as shown in Figure 11. In the following trials, the adaptive algorithms began to collect informative retraining data, gradually updated the classifiers’ boundaries that accommodated to varied signal patterns, and finally restored the locomotion recognition performance to a similar level of accuracy as seen in the first testing session. However, due to the applied static classifier, the non-adaptive algorithm failed to recover the recognition performance. It was observed that the adaptive algorithm was able to recover the accuracy within five testing trials, which indicated that the amount of retraining data collected within five trials (~25 min) might be sufficient to re-adapt to the signal variations caused by socket don-doff effects and temperature changes.

One challenge in designing and evaluating adaptive locomotion mode recognition algorithms is the lack of conceptual measures regarding what constitutes robust prosthesis control performance. Recognition accuracy or the system error rate, as an evaluation index, has been widely used in most of the current studies in this field [8,10,14,40]. However, some literature has reported a low correlation between classification accuracy and the controllability and usability of prosthetic devices [41]. Our previous study observed that not all of the recognition errors can disturb the user’s prosthetic gait performance and some errors are even not perceivable by the user [42]. This implies that purely using LMR accuracy may not be sufficient for truly evaluating the potential of adaptation work for the control of powered prosthetic legs. Instead, we suggested using “critical errors” that disturb the prosthesis user’s walking stability to quantify the control performance and robustness, which was believed to be more functionally related. In this study, the applied adaptive approach ensured that the subject ambulated safely without inducing any gait stability disturbance throughout the whole testing sessions; whereas, without the adaptive classification strategy, the subject’s walking balance was perturbed four times, as indicated in Table 3. The number of unstable disturbances elicited by errors provides us with a better insight into the clinical meaning of the potential of applying an adaptive strategy to improve system robustness.

## 4. Limitation and Future Work

Although in this study the adaptive strategy demonstrated promise in improving the robustness of powered prosthetic leg control, several study limitations were also identified. First, we did not limit the size of the retraining data in the adaptation. A huge amount of retraining data might demand a large storage memory, increase the computational burden, and decrease the classification performance by overtraining. Although the current study did not face this challenge because only 10 testing trials were conducted, future work should explore solutions to this potential cyber problem. Second, the evaluation of adaptive algorithms in this study only involved multiple sessions within a single experimental day. Future study should investigate the system robustness over days or months on more leg amputees. Third, this study was conducted in a well-defined laboratory environment. During the experiment, the number of steps in performing each locomotion activity was similar. However, in real life, the mostly encountered terrain for lower limb amputees is probably level ground. That is to say, the input testing data for LMR becomes a between-class imbalanced problem. How well the adaptive algorithms can deal with this situation is worthy of future investigation. The adaptive classification strategy reported in this study is only one potential solution to enhance the robustness of locomotion mode recognition. Additionally, in this study, we focused on the application of several well-established adaptive classifiers. We believed that the performance of the terrain recognition system could be further improved by applying other newly developed sophisticated adaptive classifiers [43,44,45]. Finally, the evaluation of adaptive LMR algorithm was conducted on a limited number of subjects and, therefore, should be considered as preliminary. Our future work will combine the adaptive approach with other engineering solutions (e.g., a robust sensing interface and fault-tolerant control scheme), and test on lower limb amputees under more realistic environments.

## 5. Conclusions

Motivated by the needs for reliable locomotion mode recognition, we investigated the feasibility of applying an adaptive classification strategy for improving system reliability when variations of input signals occurred. Three novel adaptive algorithms were investigated and evaluated based on the data collected from able-bodied subjects and subjects with unilateral transfemoral amputations. In addition, one of the tested algorithms, i.e., the entropy-based method, was initially evaluated for online prosthesis control with human-in-the-loop. Both the offline and online results showed that the adaptive pattern classification strategy was effective in enhancing the reliability of the locomotion mode recognition algorithm when changes in the input signals occurred. Moreover, the adaptive locomotion mode recognition algorithm yielded more reliable online prosthesis control across testing sessions, compared with the recognition algorithm without adaptation. Future engineering efforts are still needed to implement and evaluate the adaptive approach in real life, and eventually make the neural control of powered artificial legs practical and reliable.

## Figures and Tables

**Table 1:** Demographic information for able-bodied subjects (AB01 and AB02) and transfemoral amputees (TF01 and TF02).

	Age	Weight (kg)	Height (cm)	Gender	Years Post-Amputation	Residual Limb Length Ratio *	Daily-Use Prosthesis
AB01	38	70.0	175.8	M	-	-	-
AB02	28	84.5	181.0	M	-	-	-
TF01	59	75.1	173.5	M	23	49%	RHEO
TF02	21	61.2	180.0	M	5	52%	Genium

Note: * Residual limb length ratio: the ratio between the length of the residual limb, which was measured from the ischial tuberosity to the distal end of the residual limb, to the length of the non-impaired side, which was measured from the ischial tuberosity to the femoral epicondyle.

**Table 2:** The percentage of missed transitions for each subject.

Number of Missed Transitions	AB01	AB02	TF01
No Adaptation	12.5%	10%	15%
Entropy-based Adaptation	5%	6.25%	3.75%
LIFT Adaptation	5%	6.25%	3.75%
TSVM Adaptation	3.75%	7.5%	7.5%

Note: The total number of the task transitions was 80 for each tested subject.

**Table 3:** The number of missed transitions and unstable disturbances.

	Adaptive Trials	Non-Adaptive Trials
Number of Missed Task Transitions	2	7
Number of Unstable Disturbance Elicited by Recognition Errors	0	4

Note: The total number of the investigated task transitions was 80 for each tested subject.
